# Goldenhar Syndrome: An Atypical Presentation With Developmental and Speech Delay

**DOI:** 10.7759/cureus.36225

**Published:** 2023-03-16

**Authors:** Srilakshmi K Jayaprakasan, Muhammad Daniyal Waheed, Saima Batool, Jorge Pimentel Campillo, Maymona E Nageye, Shaniah S Holder

**Affiliations:** 1 Paediatrics, Dr. B. R. Ambedkar Medical College and Hospital, Bengaluru, IND; 2 Internal Medicine, Foundation University Medical College, Islamabad, PAK; 3 Internal Medicine, Hameed Latif Hospital, Lahore, PAK; 4 Internal Medicine, CEDIMAT, Santo Domingo, DOM; 5 Internal Medicine, Avalon University School of Medicine, Willemstad, CUW; 6 Medicine, American University of Barbados School of Medicine, Bridgetown, BRB

**Keywords:** syndrome, congenital, speech and language delay, developmental delay, goldenhar syndrome

## Abstract

Goldenhar syndrome is a rare congenital disorder that affects the development of the craniofacial region, spine, and ears. It is characterized by a wide range of symptoms that can vary in severity and may include facial asymmetry, microtia or anotia, cleft lip or palate, vertebral anomalies, and eye abnormalities. Although the cause of Goldenhar syndrome is not fully understood, it is thought to be related to disruptions in the early embryonic development of the affected tissues. The diagnosis is typically made based on physical examination and imaging studies, and management may involve a multidisciplinary team of healthcare professionals, including geneticists, audiologists, and plastic surgeons. Treatment options depend on the specific symptoms and may include surgery, hearing aids, and speech therapy. While Goldenhar syndrome can have significant physical and functional implications for affected individuals, early detection and appropriate management can help improve outcomes and quality of life.

## Introduction

Goldenhar syndrome (GS), also known as oculo-auriculo-vertebral dysplasia, is a rare multifactorial condition characterized by a defect in the development of structures derived from the first and second branchial arches [1]. The first case of GS was reported in the 1950s, and it affects one in every 3,500 to 5,000 children today, with a male predominance [2]. This condition can range from mild to severe, unilateral to bilateral, and includes craniofacial, ocular, vertebral, and auricular abnormalities [2]. Classical features include absent or partially formed ears with middle or inner ear defects causing hearing loss; hemifacial microsomia; maxillary or mandibular hypoplasia; congenital scoliosis, which occurs in 50% of cases; and epibulbar dermoids, which may lead to visual deficits [3]. GS may also affect the visceral organs that comprise the cardiac, renal, and nervous systems [3]. Some complications of visceral involvement include seizures, tetralogy of Fallot, situs inversus, and renal hypoplasia or agenesis [3].

Most cases of this congenital condition are diagnosed at birth due to the presence of classical features. However, in some cases, patients may present atypically. Developmental delays and intellectual disability are uncommon features of GS and are seen in 5-15% of cases [4]. The underlying etiology of the neurological involvement in GS is not well known; however, it impacts the cognitive and motor functions in a child, leading to an inability to process information from their surroundings and communicate their thought processes [5]. Developmental disabilities are characterized by delayed gross and fine motor skills and delayed speech onset or decreased proficiency compared to children of the same age [6,7].

In this report, we present the case of an eight-year-old female diagnosed with GS at birth; however, she did not have the typical unilateral asymmetric craniofacial or vertebral abnormalities. Instead, she had ocular defects and developmental delays in the gross, fine, and speech milestones.

## Case presentation

An eight-year-old female diagnosed with GS at birth, born from a non-consanguineous marriage, was brought to the child psychiatry department of a tertiary care hospital with complaints of delay in developmental milestones at the age of four. The child was born at term by cesarean section, weighing 2.9 kg. She had no history of neonatal jaundice or any other cardiopulmonary complications at birth. The mother also had no antenatal complications during her pregnancy. The child’s parents were healthy, and there was no family history of genetic disorders. Her birth features included bilateral upper eyelid colobomas (left larger than right), bilateral limb dermoids and lipodermoids, and preauricular skin tags. She also had incomplete eye closure, and the globe was visible in both eyes at the time of birth. She underwent corrective surgery, which included bilateral upper lid coloboma repair with eyelid reconstruction and the excision of pre-auricular tags under general anesthesia at three months of age. After canthotomy, superior cantholysis, and direct suturing, good correction of the coloboma was achieved in the right upper lid. The larger coloboma on the left side necessitated a modified Hughes sliding flap for posterior lamina reconstruction. The skin was obtained from a pre-auricular tag after soft tissue debunking; a full-thickness graft was used for anterior reconstruction. The patient had asymmetrically shaped ears, with the right ear lobule larger than the left. She did not have any hearing loss. Genetic testing was done to rule out any chromosomal abnormalities. Hence, based on her clinical presentation, especially ocular findings, bilateral limb dermoid and lipodermoids, and preauricular skin tags, a diagnosis of GS was made. The patient had no other features typical of GS such as unilateral asymmetric facial defects, ear defects, mandibular defects, or vertebral abnormalities.

At the age of three months, the patient had a few seizures, and electroencephalography (EEG) was done. The child was sedated and asleep during most of the EEG recording, and her sleep patterns were symmetrically distributed. The EEG revealed an elliptic waveform at 8 Hz over the posterior head region. She was diagnosed with a seizure disorder. The patient was prescribed Levicetiracetam, and the frequency of her seizure episodes gradually decreased. The child had not experienced any episodes in the past 10 months. However, in the later course of her development, the patient showed delays in developmental milestones. She experienced a global delay in gross motor, fine motor, social skills, and language.

**Figure 1:**
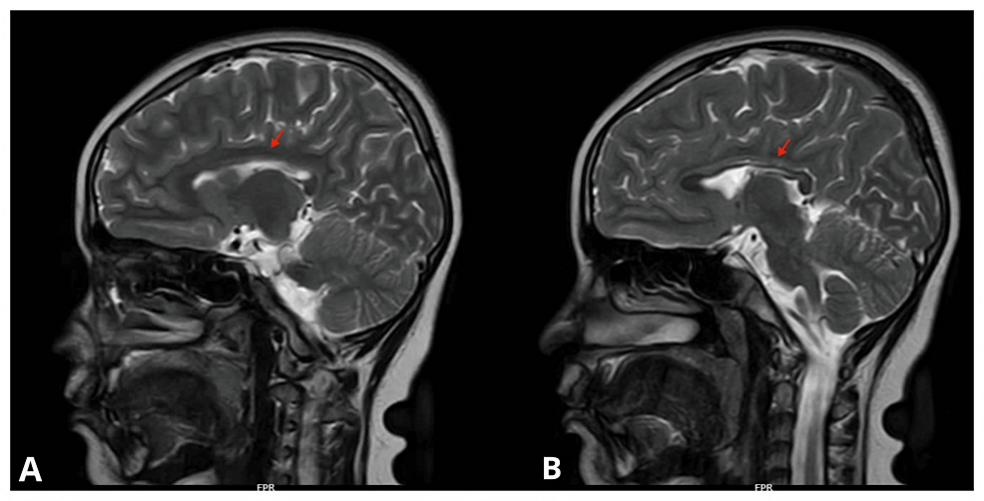
MRI of the patient showing thinned-out posterior corpus callosum (red arrow). A & B: sagittal section, sequence 4, T2-weighted imaging.

The patient was brought to the child psychiatry department of a tertiary care hospital at the age of four with complaints of delays in milestones. At the age of four, MRI was done due to delayed milestones and frequent seizure episodes, revealing a focal area of thinning of the posterior body of the corpus callosum (CC) (Figure 1).

**Figure 2:**
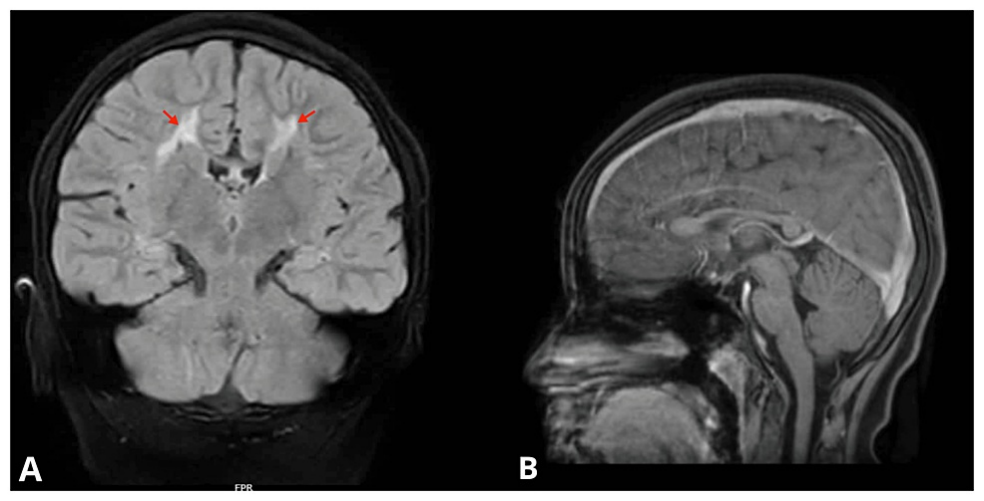
Symmetric gliotic signal with T2-FLAIR hyperintense signal (A) (coronal view) and T1 hypointense signal (B) (sagittal view). A: T2-weighted FLAIR, sequence 10. B: Sequence 15, T1-weighted. FLAIR: fluid-attenuated inversion recovery

There was a symmetric gliotic signal with a T2 fluid-attenuated inversion recovery (FLAIR) hyperintense signal and a T1 hypointense signal in both the centrum semiovale and the posterior corona radiata and peri-trigonal white matter (Figure 2).

The angular margins of the trigones of the lateral ventricles were noted. The mild gliotic signal in the right dentate nucleus was greater than that in the left dentate nucleus. A thinned-out posterior corpus callosum was suspected to be causing a delay in cognitive and speech development. No radiological abnormalities were found on the cervical and spinal X-rays. The patient had two siblings with normal development. Currently, the child is receiving treatment for a delay in speech, gross, and fine motor skills. It includes speech and language therapy along with occupational therapy. She is also on quetiapine for her mood changes, in addition to the antiepileptics. No significant improvement in speech has been observed with speech and language training. She hoots and makes sounds but cannot say or write words. Her vocabulary is limited to single words in the native language, indicating the names of her mother and father, which are also not proper or complete. Gross motor skills have shown improvement with occupational therapy, which is mostly activity-oriented. The child is presently attending a school for special children affiliated with the psychiatry department of the hospital. However, she still lacks social cues and finds it difficult to be around her peers at the special school as well.

## Discussion

The branchial arches form within the fourth week of development and are responsible for developing many craniofacial structures [7]. The first brachial arch forms the mandible, incus, malleus, muscles of mastication, and the maxillary and mandibular branches of the trigeminal nerve [8]. The second brachial arch produces the body and lesser horn of the hyoid, the stapes in the ear, the muscles of facial expression, and the facial nerve [8]. Abnormal development of these structures characterizes the condition known as GS and may be caused by mesodermal or neural crest cell migration disruption or abnormal embryonic vascular supply to the branchial arches during fetogenesis [1]. The exact genetic etiology of GS is unknown; however, some studies suggest that this condition may be due to a possible underlying chromosomal abnormality that may arise sporadically or be inherited [9]. The risk of developing GS is higher in children with maternal use of teratogenic substances such as retinoic acid, tamoxifen, cocaine, and alcohol during pregnancy or with prenatal infection with rubella or influenza [1].

GS is characterized by the classic triad of mandibular hypoplasia, ocular and auricular malformations, and vertebral abnormalities [10]. However, there have been increasing reports of children presenting without all of the classical symptoms. Some additional features reported include developmental delays and multiple complex odontomas [11]. There is a paucity of literature exploring the association between GS and developmental delay, with most studies being case reports; therefore, the underlying cause is still being determined. Some typical cerebral findings in GS include Arnold-Chiari malformations, frontal lobe hypoplasia, and encephalocele; however, these features were absent in this case [12]. Due to the various cerebral alterations caused by GS, involvement of the corpus callosum may occur as well.

The CC is a thick bundle of nerve fibers that connects the two cerebral hemispheres, allowing them to communicate and coordinate appropriate bodily functions [13]. There is a possibility that GS may affect this structure, leading to gliosis and malformation or destruction of the CC. The severity of CC involvement in any congenital disease ranges from mild thinning to hypoplasia to agenesis in severe cases [14]. A case by Trivedi et al. reported agenesis of the CC found on CT in a five-year-old child with GS who had a history of upper lid coloboma, limbal dermoids, multiple skin tags, and developmental delay without the classic symptoms such as mandibular hypoplasia or vertebral abnormalities [4]. Embryologically, the CC develops in an anterior-to-posterior direction except for the rostrum, which is formed last [15]. The thinning of the CC is differentiated into primary and secondary. Primary thinning is due to defective myelination during development, as is seen in microcephaly, some metabolic disorders, and leukoencephalopathies [16]. It is characterized by the presence of the anterior structures of the CC and the thinning or absence of the posterior structures [15,16]. Secondary thinning of the CC is characterized by dysmyelination and focal thinning of structures in either the anterior or posterior areas of the CC after development, leading to callosal anomalies [16]. In this case, the rostrum was entirely developed, and there was localized thinning of the posterior body, indicating secondary thinning of the CC.

In this case, thinning of the CC was possibly due to cerebral gliosis. Gliosis occurs when there is an injury to the central nervous system due to infarcts, infections, toxins, or neoplasms, which may lead to scarring and cerebral dysfunction [17]. On MRI, gliosis is characterized by an increased T2/FLAIR signal and a reduced T1 signal, signifying areas of demyelination and axonal loss [17]. This patient had a gliotic signal in multiple areas of the brain. Due to the underlying demyelination associated with gliosis, involvement of the CC may precipitate secondary thinning. The exact mechanism of how GS leads to gliosis and subsequent thinning of the CC is unknown and requires further research. However, due to the patient’s age and the lack of alternative findings such as brain tumors, infections, or cerebral ischemia, it is theorized that GS was the primary cause of the gliosis and callosal thinning that led to her symptoms of developmental delay and seizures.

The diagnosis of GS is clinical. Imaging tests such as a CT scan, an MRI, an ultrasound, an echocardiogram, and an EEG can be conducted to assess the severity of symptoms [18]. In this case, our patient had a history of bilateral colobomas, limbal dermoids, and preauricular skin tags, minus the common symptoms such as craniofacial defects or vertebral abnormalities. EEG results in the patient were unremarkable, with normal brain sleep waves and theta waves in the occipital region when the child was awakening. This was possibly a result of changing sleep activity and is commonly seen in children and adolescents [19]. The brain MRI results in this patient included gliosis of the centrum semiovale, the posterior corona radiata, and peri-trigonal white matter, as well as thinning of the posterior body of the CC. Other MRI findings associated with GS include widening of the foramen of Magendie and the cisterna magna, non-visualization of the external auditory canal, and absence of the lateral semicircular canal [20].

Treatment of GS is symptomatic and may include a combination of supportive, medical, and surgical modes of therapy. Feeding assistance and hearing aids and glasses for visual and hearing impairments are commonly used [18]. Physical and speech therapy show great promise in the improvement of developmental delays, and cosmetic surgery, including mandibular reconstruction, dermoid excisions, and cleft lip and palate repair, is done in patients to improve feeding and their overall general appearance [1]. In our case, the patient was managed medically with levetiracetam for her seizure disorder, and speech and occupational therapy were implemented to improve her language and motor skills, the latter of which was greatly improved.

It is essential for physicians to note that while most patients present classically, a few cases are atypical; therefore, a high index of clinical suspicion is required. This will allow for early diagnosis and management of GS and prevent a decline in the child’s quality of life.

## Conclusions

GS is a rare multifactorial condition possibly caused by defective cellular migration and is characterized by the abnormal development of the structures derived from the first and second branchial arches. It is classically characterized by the triad of mandibular hypoplasia, ocular and auricular malformations, and vertebral abnormalities. The effects of GS on the brain are not well known; however, there is vast neurological involvement associated with this syndrome that requires further research. In this report, it is suspected that the global developmental delay and seizures were caused by focal thinning of the CC and surrounding cerebral hemispheres due to underlying gliosis. Physicians should be aware of this possible association and implement diagnostic tests such as MRI when presented with children with developmental delay and seizures in the setting of GS. Early diagnosis and intervention with symptomatic treatment and psychosocial support will improve the quality of life for children as they age and adjust to functioning in society.
